# Molecular Changes Induced by Oxidative Stress that Impair Human Sperm Motility

**DOI:** 10.3390/antiox9020134

**Published:** 2020-02-04

**Authors:** Karolina Nowicka-Bauer, Brett Nixon

**Affiliations:** 1 Institute of Human Genetics, Polish Academy of Sciences, 60-479 Poznan, Poland; 2 Priority Research Centre for Reproductive Science, School of Environmental and Life Sciences, Discipline of Biological Sciences, University of Newcastle, Callaghan, Newcastle, NSW 2308, Australia; brett.nixon@newcastle.edu.au; 3 Hunter Medical Research Institute, Pregnancy and Reproduction Program, New Lambton Heights, Newcastle, NSW 2305, Australia

**Keywords:** 4-hydroxynonenal (4HNE), infertility, lipid peroxidation, male germ cells, oxidative stress, reactive oxygen species, spermatozoa, sperm capacitation, sperm motility

## Abstract

A state of oxidative stress (OS) and the presence of reactive oxygen species (ROS) in the male reproductive tract are strongly correlated with infertility. While physiological levels of ROS are necessary for normal sperm functioning, elevated ROS production can overwhelm the cell’s limited antioxidant defenses leading to dysfunction and loss of fertilizing potential. Among the deleterious pleiotropic impacts arising from OS, sperm motility appears to be particularly vulnerable. Here, we present a mechanistic account for how OS contributes to altered sperm motility profiles. In our model, it is suggested that the abundant polyunsaturated fatty acids (PUFAs) residing in the sperm membrane serve to sensitize the male germ cell to ROS attack by virtue of their ability to act as substrates for lipid peroxidation (LPO) cascades. Upon initiation, LPO leads to dramatic remodeling of the composition and biophysical properties of sperm membranes and, in the case of the mitochondria, this manifests in a dissipation of membrane potential, electron leakage, increased ROS production and reduced capacity for energy production. This situation is exacerbated by the production of cytotoxic LPO byproducts such as 4-hydroxynonenal, which dysregulate molecules associated with sperm bioenergetic pathways as well as the structural and signaling components of the motility apparatus. The impact of ROS also extends to lesions in the paternal genome, as is commonly seen in the defective spermatozoa of asthenozoospermic males. Concluding, the presence of OS in the male reproductive tract is strongly and positively correlated with reduced sperm motility and fertilizing potential, thus providing a rational target for the development of new therapeutic interventions.

## 1. Introduction

Male infertility accounts for approximately 40% of all cases of infertility [1] and affects approximately 7% of all men worldwide [2]. Recently, considerable attention has focused on the role of oxidative stress (OS) in the pathophysiology of male infertility. OS is associated with the excessive generation of free radicals, such as reactive oxygen species (ROS), and/or decreased efficacy of antioxidant defenses. In humans, the pervasive impact of OS has been linked to the development of a variety of diseases as diverse as Alzheimer’s disease [3], cancer [4], heart failure [5] and obesity [6]. OS is also a prevalent biomarker associated with the semen of approximately 35% of infertile men [7] and the presence of elevated levels of seminal ROS has been reported arising from male reproductive tract pathologies such as varicocele [8], inflammatory [9] and prostate cancer [10]. Owing to their unique architecture, featuring an abundance of oxidizable substrates and limited intracellular antioxidant defenses, the male germ cell is particularly vulnerable to elevated levels of ROS. The resultant OS commonly manifests in a spectrum of adverse sequelae, which drive germ cell dysfunction and culminate in their apoptotic demise [11]. Accordingly, patients with increased ROS levels in their seminal plasma [12] often present with reduced sperm count (oligozoospermia); a condition attributed, at least in part, to apoptosis within the developing germ cell population [13,14]. However, in addition to the loss of sperm viability, OS has also been causally linked to lesions in the motility profile of mature spermatozoa [15].

Progressive sperm movement is required for delivery of the male gamete to the site of fertilization within the ampulla of the Fallopian tubes, as well as the resultant syngamy that facilitates transfer of paternal genetic and epigenetic information to an oocyte during natural conception. Effective sperm motility also plays a crucial role during the assisted reproduction technology (ART) procedures of intrauterine insemination and in vitro fertilization. According to World Health Organization (WHO) criteria [16], the presence of less than 32% of spermatozoa with progressive motility in an ejaculate is defined as asthenozoospermia. It is estimated that asthenozoospermia accounts for as much as 30% of all cases of male infertility [17] and, in the absence of genetic defects, bacterial infection or abnormal semen liquefaction, this condition is often directly linked to the presence of OS in the male reproductive tract and semen [18]. Indeed, elevated levels of seminal ROS have repeatedly been documented in studies of astheno- and oligoasthenozoospermic individuals [18,19,20,21]. In terms of the mechanistic basis of sperm dysfunction leading to asthenozoospermia, defective mitochondria and a concomitant reduction in the production of energy required to support normal movement have been identified as a common etiology [18]. However, structural changes in the motility apparatus housed within the sperm flagellum and dysregulation of motility-associated signaling pathways have also been reported in response to OS [22], thus complicating the diagnosis of sperm motility defects. Here, we survey the literature pertaining to the generation of ROS in the male reproductive tract and the deleterious influence of OS on the biochemical pathways and structural features of the sperm cell responsible for modulating their motility. We also highlight the role of antioxidants in combating the burden of OS associated infertility.

## 2. Sources of ROS in Semen

Seminal ROS originate from a variety of different endogenous and exogenous sources. In addition to generation by spermatozoa themselves, other cellular contaminants such as immature round germ cells, leukocytes and epithelial cells, can also have a direct bearing on the levels of ROS within an ejaculate. Additionally, a number of environmental and lifestyle factors can exert direct and indirect influence over the levels of OS encountered within the male reproductive tract.

### 2.1. Endogenous Sources of ROS

#### 2.1.1. Spermatozoa

At least two distinct pathways have been implicated in ROS generation in mature spermatozoa. One such pathway is localized within the sperm plasma membrane and is linked to the activity of the nicotinamide adenine dinucleotide phosphate (NADPH) oxidase system, whilst the second is associated with electron leakage from the mitochondrial electron transport chain (ETC) [23]. The latter of these pathways represents the main source of ROS production in spermatozoa and occurs as a result of the premature exit of electrons from the respiratory chain. This leakage prevents the reduction of oxygen to water at cytochrome c oxidase, with the escaped electrons instead reacting with molecular oxygen (O_2_) to form the superoxide radical (O_2_•^−^). Basal levels of electron leakage can potentially occur from multiple sites within the ETC (reviewed in [24]), and, unlike the O_2_•^−^ generated at the level of the plasma membrane that supports sperm capacitation [25], mitochondrial derived O_2_•^−^ is generally associated with pathological damage. Indeed, when superoxide anion generation exceeds the limited antioxidant capacity of the sperm cell, it has the ability to propagate the formation of non-radicals, including hydrogen peroxide (H_2_O_2_), and in the presence of Fe^3+^, the formation of alternative radicals such the hydroxyl anion (OH^−^) via Haber-Weiss and Fenton reactions [26]. Additional ROS containing nitrogen atoms (such as NO, NO_3_^−^, NO^−^, N_2_O, and HNO_3_) are also able to be formed [27,28] and collectively, these powerful oxidants have the potential to trigger the peroxidation of membrane lipids and a concomitant loss of sperm function [29]. In studies conducted in rats it has been shown that the presence of dead spermatozoa can also promote higher than normal levels of H_2_O_2_ [30]. This response appears to be related to the apoptosis cascade during which the disruption of mitochondrial membranes leads to the release of cytochrome c and elevated ROS production [31].

#### 2.1.2. Immature Germ Cells

In addition to mature spermatozoa, ejaculated semen samples also frequently contain variable amounts of contaminating immature sperm cells, which have failed to complete normal morphological differentiation during spermatogenesis [32]. Depending on the timing of such errors, they may result in the presence of either round cells or seemingly mature spermatozoa that retain a considerable portion of their cytoplasm. The latter cells originate from defects encountered in the final phase of spermatogenesis (i.e., spermiogenesis), during which the majority of the cell’s cytoplasm would otherwise be shed to create the highly streamlined profile of the sperm head [33]. The residual cytoplasm retained in these immature sperm cells tends to accumulate in the vicinity of the mid-piece and contains high levels of glucose-6-phosphate dehydrogenase; an enzyme that catalyzes NADPH production via the pentose phosphate pathway [34]. NADPH, in turn, is capable of acting as a substrate to fuel ROS-generating NADPH oxidases. Thus, the presence of immature germ cells harboring excess cytoplasm has the potential to elevate endogenous ROS levels within an ejaculate and deleteriously affect the function of their otherwise normal counterparts [34].

#### 2.1.3. Leukocytes

A state of infection and/or inflammation in the male reproductive tract (i.e., testis, epididymis, seminal vesicles and/or prostate) can result in increased infiltration of leukocytes bringing with them an attendant risk of elevated ROS within an ejaculate [35]. Indeed, when appropriately stimulated, phagocytic leukocytes are capable of metabolizing oxygen to produce copious quantities of ROS in a process often referred to as a respiratory burst [36]. This microbicidal defense response is mediated by the NADPH-oxidase complex, and enhanced by the presence of cytokines, which themselves are released during inflammation [9,37]. It follows that the presence of leukocytes, and predominantly polymorphonuclear neutrophils, is recognized as a major source of ROS in human semen [37,38].

#### 2.1.4. Varicocele

Varicocele is a pathology associated with an abnormal enlargement of the pampiniform venous plexus surrounding the spermatic cord in the scrotum [39]. This clinical condition occurs in approximately 15% of males and accordingly represents the most common cause of primary and secondary infertility in men; accounting for as much as 40% of all cases of male infertility [40]. Consistent with this clear association between varicocele and male infertility, a considerable body of evidence supports OS as a key causative agent in the pathophysiology of varicocele [41]. Indeed, both infertile patients and fertile men with varicocele frequently present with higher levels of ROS, NO, and lipid peroxidation products in their reproductive tract than men without varicocele [42,43,44,45,46]. The most widely accepted model to account for these findings suggests that varicoceles lead to an increase in scrotal temperature owing to the reflux and accumulation of warmed abdominal blood within the pampiniform plexus. The resultant heat stress has, in turn, been postulated to enhance ETC electron leakage via thermal inhibition of mitochondrial complexes, thus accentuating mitochondrial ROS production (reviewed in [47]).

## 3. Exogenous Sources of ROS

In addition to endogenous influences, modern lifestyle and environment factors have also been increasingly linked with a range of adverse health sequelae, including poorer quality semen parameters. Below, we give brief consideration to physical and chemical factors that have been associated with heightened levels of seminal ROS.

### 3.1. Physical Factors

One of the contemporary issues of the modern lifestyle is excess heating of testes. In scrotal animals such as our own, the testicular environment has evolved to operate optimally at temperatures that generally lie at least 1–2 °C below that of core body temperature (reviewed in [48]). Accordingly, the spermatogenic process can be adversely impacted by scrotal hyperthermia as occurs in certain professions such as those that involve extended periods of seated activity (e.g., at a desk or in a vehicle) or those that involve direct exposure to high ambient temperatures (e.g., steel fabrication and welding). The occupational practice of using a laptop computer in such a way that it covers the testes can also cause localized heat stress, as does the wearing of tight clothing and in particular, underwear. Through mechanisms described in the previous section, the heat-stressed testes have been shown to produce excessive ROS, which is linked to the impairment of sperm function [49,50]. It should be noted that alternative lifestyle habits such as frequent sauna or warm bath exposures also represent potential sources of heat stress that may impact on male infertility [51,52,53]. In support of this conclusion, a recent study by Houston et al. [54] demonstrated that the exposure of mice to elevated ambient temperature led to increased sperm mitochondrial ROS generation and OS-induced molecular changes during germ cell development.

Similarly, increasing attention has been focused on the potential impact of non-ionizing radiation, such as the electromagnetic energy (EME) emitted by mobile technologies and other forms of microwave equipment, on the male germ line [55]. In this context, the human body has the potential to behave in a manner analogous to that of an antenna that receives EME [56]. The exposure of human tissues to EME can have various biological effects including localized elevation of the temperature in the affected tissue, including the testes [56]. EME can also alter cellular membrane potential and impact molecular bonding, with the polar side chains of amino acids being particularly affected by EME exposure [57]. Such changes not only have the potential to influence protein structure, and hence interfere with enzymatic activity, but can also perturb the transmembrane transport of ions [56,57]. As an extension of these findings, multiple studies have now demonstrated that supraphysiological levels of EME can negatively affect mitochondrial membrane characteristics and/or overall functioning [58,59,60]. Indeed, the exposure of isolated cells to EME can lead to increased activation of mitochondrial function and an attendant elevation of ROS production associated with complexes I and III of ETC, independent of changes in mitochondrial membrane potential [56,58]. In model cells such as those of the human amniotic epithelial lineage, magnetic fields can induce mitochondrial permeability transition and cytochrome-c release together with increased intracellular ROS generation, via a pathway that is dependent on glycogen synthase kinase-3β [58]. As proof of concept, it has also been shown that human spermatozoa exposed to EME at frequencies designed to simulate that emitted by mobile phones experience reduced motility and vitality; defects that were associated with increased mitochondrial ROS production and numerous molecular alterations that are synonymous with OS [59].

### 3.2. Chemical Factors

Aside from the physical factors discussed above, male fertility is also sensitive to a variety of toxicants, such as those arising from industrial processes or from common everyday materials, which accumulate in the human body. Indeed, it is well established that the accumulation of the heavy metals lead and cadmium can impair multiple semen parameters, including sperm motility [61]. Similarly, male rats treated with lithium display OS in their testes and experience reduced sperm count and motility [62]. The induction of OS in the male reproductive tract has also been cited as a causal agent responsible for elevated levels of apoptosis among developing germ cells, defects in sperm morphology and impaired sperm function in mice treated with industrial contaminants used in the production of plastics, such as bisphenol-A [63], mono-butyl phthalate [64] and other related compounds [65]. This spectrum of deleterious OS-related effects extends to other forms of chemical exposure including those associated with excessive alcohol consumption or cigarette smoking. In this context, chemicals contained within cigarette smoke have been shown to cause local inflammation, an attendant 48% increase in seminal leukocytes, and a 107% increase in seminal ROS levels [66]. It follows that the semen of cigarette smokers is not only characterized by increased ROS levels, but also extensive molecular changes in the spermatozoa and reduced overall semen quality [67]. Likewise, excessive alcohol consumption can lead to ethanol-induced cell membrane destruction, increased production of ROS and impaired sperm function [68,69].

**Figure 1:**
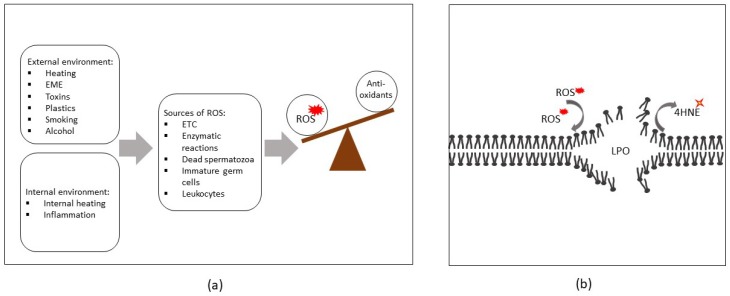
(**a**) Sources of reactive oxygen species (ROS) in human spermatozoa and the relationship between the rate of their production and antioxidant defenses during oxidative stress. (**b**) ROS are capable of attacking polyunsaturated fatty acids (PUFAs) within cellular membranes, initiating lipid peroxidation cascades (LPO) and resulting in the production of cytotoxic lipid aldehydes such as 4-hydroxynonenal (4HNE). Abbreviations: EME, electromagnetic energy; ETC, electron transport chain.

From the preceding discussion, it is apparent that acute and/or chronic exposure to a variety of external or internal factors can trigger the overproduction of ROS and reduce antioxidant defenses within the male reproductive tract, thus propagating an OS cascade and resulting in LPO (summarized in Figure 1).

## 4. ROS-Induced Lesions Detected in Low-Motility Spermatozoa

With the diverse range of factors that can amplify the levels of ROS in semen, attention has naturally focused on the impact of these powerful oxidants on sperm function. Through decades of research we have come to realize that the highly specialized sperm cell is exceptionally vulnerable to disturbance in ROS levels owing to the presence of modest antioxidant defenses and conversely, a myriad of oxidizable substrates [70]. Not the least of these are the polyunsaturated fatty acids (PUFAs; such as linolenic, arachidonic and decohexaenoic acids) that dominate the lipid architecture of the sperm plasma membrane. In the case of human spermatozoa, the predominant PUFA is decohexaenoic acid (DHA), a lipid that accounts for >50% of all membrane PUFAs [70,71]. PUFAs such as DHA not only play a major role in the regulation of sperm membrane fluidity, but owing to the presence of multiple carbon-carbon double bonds, they also serve as prime substrates for ROS attack. The resultant cascade of lipid peroxidation (LPO) reactions catalyze the formation of numerous breakdown products including a suite of highly reactive lipid aldehydes [e.g., malondialdehyde (MDA), 4-hydroxynonenal (4HNE) and acrolein] [11] (see Figure 1b).

Under physiological conditions, aldehyde-metabolizing enzymes function to detoxify and prevent the accumulation of these advanced end products of LPO [72]. However, excessive OS can promote the accrual of lipid aldehydes and, owing to their inherent stability (relative to that of free radicals), these electrophiles can elicit widespread cellular damage and pathological dysfunction in human spermatozoa [73,74]. In the case of 4HNE, which ranks among the most abundant and cytotoxic of the lipid aldehydes, the chemical structure contains three reactive functional groups: a C2=C3 double bond, a C1=O carbonyl group and a hydroxyl group on C4 [75]. These structural elements render the 4HNE electrophile highly reactive toward nucleophilic groups, enabling the formation of both the Michael addition of thiol or amino compounds and Schiff bases with primary amines. Thus, 4HNE has the ability to react with proteins (principally those containing histidine, cysteine and lysine residues), lipids, and nucleic acids (mostly with the guanosine moiety of DNA) [76,77,78]. In spermatozoa, the creation of 4HNE adducts has been linked with compromised membrane integrity, motility defects and reduced ability to participate in oocyte interactions [22,73,79]; thus reducing overall fertility. Moreover, studies by Bromfield and colleagues [73,79] have shown that the impact of 4HNE can vary depending on the timing and of the insult. Thus, 4HNE can drive post-meiotic round spermatids towards a ferroptotic cell death pathway, whereas an equivalent exposure of mature spermatozoa can elicit functional lesions, which compromise their fertilization potential (e.g., dysregulation of the molecular chaperone Heat Shock Protein A2 and an accompanying disruption of oocyte recognition), but does not overtly impact their viability [73,79]. Such differential pathogenesis may be attributed to the highly specialized architecture of the male germ cell, which depending on their stage of differentiation, features an abundance of substrates for 4HNE attack, minimal antioxidant defense enzymes, and limited capacity for self-repair when 4HNE-mediated damage is sustained [11]. However, excess ROS production can also directly impact sperm function via increases in redox driven protein modifications [80]. Thus, OS has been shown to promote an increase in S-glutathionylation and tyrosine nitration of sperm proteins, both of which adversely impact motility [80,81]. Similar alterations have been documented by Vignini et al. [82], who demonstrated an increase in ONOO^−^ concentrations and tyrosine nitration in human asthenozoospermic sperm samples.

### 4.1. Compromised Sperm Membrane Integrity

The peroxidation of membrane lipids leads to the catabolism of phospholipids and liberation of PUFAs, thus directly contributing to increased membrane fluidity and permeability to ions, which can elicit downstream effects in terms of inactivating membrane enzymes and receptors [20,79]. The loss of sperm membrane integrity is also tightly correlated with reduced sperm motility [83]. Indeed, the compromise of both of these parameters has been well documented in the context of cryopreservation [84,85,86,87] in which post-thaw sperm samples typically experience a burst of ROS and high levels of OS [85,86,88]. Sperm motility is also highly sensitive to pH and ion concentration (reviewed in [89]). The disruption of sperm membrane integrity alters the diffusion of ions across the membrane and dysregulates the function of ion pumps and channels, especially as those ion channels that are regulated by PUFAs (reviewed in [90]). Although there is currently limited direct evidence linking sperm membrane integrity and ion channel function, the peroxidation of lipids and the downregulation of some ion channels in low-motility spermatozoa has been documented [83,91,92,93,94]. In this context, Baker et al. [22] reported two sperm ion channels, voltage-dependent anion-selective channel protein 2 (VDAC2) and sodium/potassium-transporting ATPase subunit alpha-4 (ATP1A4) that are susceptible to the formation of potentially deleterious adducts with 4HNE. Similarly, decreased VDAC and ATP1A4 abundance has been reported in the spermatozoa of males with asthenozoospermia [95] and in patients with unilateral varicocele (a pathology correlated with OS) [96], respectively.

The deleterious impact of ROS extends beyond the plasma membrane to also include those membranes surrounding subcellular organelles such as the mitochondria. In contrast to somatic cells, sperm mitochondria are localized in one specific region of the sperm cell—the mid-piece of the flagellum. Within this domain, the mitochondria are organized end to end to form long spirals that wrap tightly around the axoneme [97,98]. The correct functioning of mitochondria is especially important for sperm cells in terms of supplying the energy needed for efficient movement. The elevated production of mitochondrial ROS and the resultant propagation of sperm plasma membrane LPO can, in turn, initiate secondary LPO in mitochondrial membranes. Similar to the plasma membrane, the peroxidation of lipids residing in the mitochondrial membranes also changes the fluidity of these structures, thus resulting in the upregulation of proton and electron leakage through the inner membrane [99,100]. Such a situation leads to the loss of mitochondrial membrane potential (∆Ѱ), compromises the efficiency of mitochondrial ATP generation, and triggers a cycle of elevated electron leakage and the generation of additional mitochondrial ROS, which collectively exacerbates the impact of OS on sperm function [18,101,102].

### 4.2. Dysregualtion of Sperm Metabolic Enzymes 

In human sperm cells, the majority of the energy needed to support motility appears to originate from glycolysis, which takes place in the sperm tail. However, it has been argued that mitochondrial oxidative phosphorylation (OXPHOS) also plays a secondary role in the bioenergetic pathways associated with motility. In this context, a key substrate for OXPHOS are the endogenous lipids present in the sperm membrane [103], but other metabolic substrates and their associated pathways have also been implicated [18,103,104]. Indeed, depending on the prevailing environmental conditions that sperm encounter on route to the site of fertilization, these cells may be able to utilize different metabolic pathways.

**Figure 2:**
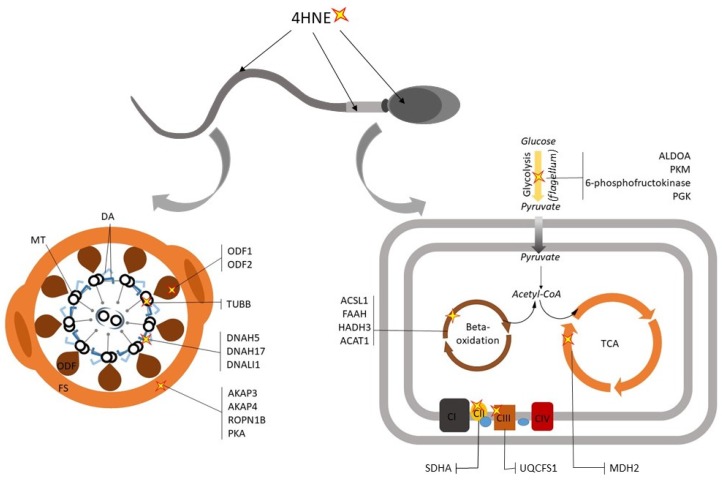
The influence of 4-hydroxynonenol (4HNE) on sperm function. The lipid aldehyde 4HNE has repeatedly been shown to form adducts with sperm flagellum proteins associated with the motility apparatus, signaling pathways and metabolism. In the context of the mitochondria, 4HNE adduction has been linked to adverse effects on enzymes of beta-oxidation, the tricarboxylic acid (TCA) cycle and the electron transport chain (ETC), thus attenuating the energy production. Abbreviations: CI, ETC Complex I; CII, ETC Complex II; CIII, ETC Complex III; CIV, ETC Complex IV; DA, dynein arms; FS, fibrous sheath; MT, microtubules; ODF, outer dense fibers.

As previously mentioned, the cytotoxic aldehydes generated during LPO can form adducts with multiple elements of the sperm proteome. Curiously, however, not all sperm proteins appear to display equivalent susceptibility to lipid aldehyde carbonylation reactions [105]. By way of example, the application of affinity-based isolation techniques coupled with mass spectrometry has revealed that 4HNE preferentially adducts to a relatively small number of putative targets in human spermatozoa [22]. Notably, many of these 4HNE targets serve as metabolic enzymes responsible for the production of energy needed to support sperm motility (depicted in Figure 2), including several glycolytic enzymes [phosphofructokinase (PFKP); aldolase A, fructose-bisphosphate (ALDOA); phosphoglycerate kinase (PGK), pyruvate kinase (PKM); lactate dehydrogenase C chain (LDHC)], enzymes involved in the TCA cycle [malate dehydrogenase 2, NAD (mitochondrial) (MDH2)] [22], electron transport chain [succinate dehydrogenase complex, subunit A, flavoprotein (SDHA) [106]; ubiquinol-cytochrome c reductase, Rieske iron-sulfur polypeptide 1 (UQCRFS1)] [22] and beta-oxidation [fatty acid amide hydrolase (FAAH); acyl-CoA synthetase long-chain family member 1 (ACSL1); hydroxyacyl-CoA dehydrogenase trifunctional multienzyme complex subunit beta (HADHB); acetyl-Coenzyme A acetyltransferase 1 (ACAT1)] [22]. Moreover, 4HNE has also been found to adduct with solute carrier family 25 (mitochondrial carrier; adenine nucleotide translocator), member 31 (SLC25A31), a mitochondrial carrier protein involved in the exchange of cytoplasmic ADP with mitochondrial ATP [22]. Although the consequences of damage caused by the insertion of bulky 4HNE (C-9) carbonyl adducts has yet to be investigated across all targeted sperm proteins, it is reasonable to suspect that these modifications could elicit protein mis-folding, poor substrate recognition, and/or degradation of the protein itself [107,108]. Indeed, recent work by Aitken et al. [106] has demonstrated that 4HNE adduction to SDHA can activate mitochondrial electron leakage and disrupt ∆Ѱ in human spermatozoa. These data raise the prospect that 4HNE may primarily perturb the bioenergetic pathways that sustain sperm motility and thus provide a mechanistic model to account for the dysregulation of sperm function commonly reported in cells burdened by excessive ROS production [68]. Notably, the self-perpetuating nature of this pathway may also account for the ability of 4HNE to drive human spermatozoa towards an intrinsic apoptotic pathway.

Such a model takes on added significance in view of proteomic data emerging from studies of the spermatozoa of asthenozoospermic patients. Whilst not universal, a common theme to emerge from this work is that proteins involved in energy production are generally downregulated in the defective spermatozoa of asthenozoospermic individuals [18,105,109]. Indeed, the majority of proteins so affected in asthenozoospermia are linked to glycolysis, pyruvate metabolism, tricarboxylic acid cycle (TCA), OXPHOS, beta-oxidation or alternative metabolic pathways [18,109,110]. By way of example, the glycolytic enzyme, glucose-6-phosphate isomerase (GPI), has been shown to be under-represented in asthenozoospermic individuals, as has the sperm-specific glyceraldehyde-3-phosphate dehydrogenase (GAPDHS) [110,111,112,113]; the activity of which is also attenuated after treatment of spermatozoa with ROS [112]. Moreover, several mitochondrial enzymes involved in the downstream conversion of pyruvate to acetyl-CoA, TCA cycle, ETC and a plethora of ATP synthase subunits have also been shown to be dysregulated. Similar reductions in protein abundance also extend to several enzymes involved in beta-oxidation of fatty acids [18,95,105,109,110]. Alternative proteomic studies of asthenozoospermia have revealed downregulation of one of the subunits of NADH dehydrogenase (NDUFA13), a key constituent of complex I of the ETC [114]. The knockdown of this protein in a mouse spermatocyte cell line led to the loss of ∆Ѱ, increased ROS production and apoptosis [114]. These data draw interesting parallels with the study of asthenozoospermic individuals by Nowicka-Bauer et al. [18], which clearly showed the downregulation of sperm mitochondrial metabolic pathways and ∆Ѱ; defects that were accompanied by increased production of mitochondrial ROS in the spermatozoa of asthenozoospermic patients. These data reinforce the notion that reduced sperm motility is predominantly associated with dysfunction of sperm mitochondria leading to elevated levels of ROS and a reciprocal reduction in energy production. However, it remains to be established what factor(s) are responsible for the under-representation of metabolic enzymes in the spermatozoa of asthenozoospermic individuals and whether this is in any way linked to localized ROS generation within the vicinity of the developing germ cells.

### 4.3. Defects in the Sperm Motility Apparatus and Signaling Pathways

The flagellum, which is responsible for the propagation of sperm motility, is constructed around a cytoskeletal structure known as the axoneme. The axoneme, in turn, consists of tubulin microfilaments arranged in a highly conserved 9 + 2 scheme (whereby 9 doublets surround one central pair). To each doublet of microtubules are attached two dynein arms (outer and inner), which act as motor proteins capable of ‘walking’ along the adjacent microtubules and effecting the sliding of microtubules relative to each other (reviewed in [115]). This creates an undulatory wave that is propagated along the length of the flagellum to generate the rhythmic beating patterns responsible for propelling sperm forward [116]. Aside from the core elements described above, the sperm axoneme is also supported by a family of accessory proteins known as outer dense fibers (ODF1–ODF4), which uniquely among mammalian spermatozoa, are localized around the microtubules and provide additional elasticity and stability to flagellum movement [117]. Given the fundamental importance of motility in terms of delivering spermatozoa to the site of fertilization, it follows that any defects in the axonemal structure, or the ODF accessory proteins, can compromise fertility [118]. Accordingly, several studies on asthenozoospermia have linked this condition with an attendant change in the levels of structural proteins residing in the flagellum. Among the most frequently reported of these are the dynein motor proteins, ODFs and those belonging to the tektin family of microtubule-stabilizing proteins [18,109,117].

In a similar context, studies on sperm protein adducts arising from direct 4HNE challenge have demonstrated that this aldehyde can readily bind to tubulin (TUBB), several members of the dynein family (DNAH5, DNAH17, DNALI1), and the ODF1 and ODF2 proteins [22] (see Figure 2). Notably, these findings align well with the proteomic deficits identified in the spermatozoa of asthenozoospermic individuals [18,109,110]. As an extension of this work, however, it has also been shown that core elements of the sperm fibrous sheath (a unique cytoskeletal structure that surrounds the axoneme and outer dense fibers and regulates the flexibility and shape of the flagellar beat [119]) are highly sensitive to 4HNE adduction. In particular, the major fibrous sheath components of A-Kinase Anchoring Protein (AKAP4 and AKAP3) and Rhophilin Associated Tail Protein 1B (ROPN1B), which contribute to the structural organization of the fibrous sheath [119,120], have been identified as dominant 4HNE targets [22,99,101]. Indeed, AKAP4 has recently been validated as a conserved target for 4HNE adduction in primary cultures of post-meiotic male germ cells (round spermatids) and in mature mouse and human spermatozoa [121]. Through the application of an exogenous 4HNE treatment regimen, we further demonstrated that 4HNE modification resulted in a substantial reduction in the levels of AKAP4 detected in round spermatids and mature spermatozoa alike. Moreover, reduced AKAP4 levels were correlated with dysregulation of the capacitation-associated signaling framework assembled around the AKAP4 scaffold.

These data accord with the demonstration that the spermatozoa of male mice lacking AKAP4 display reduced progressive movement and are infertile [122]. Similarly, reduced levels of AKAP4 have also been implicated as a biomarker of human asthenozoospermic males [95]. In addition to AKAP4, alternative cAMP-responsive elements such as protein kinase A (PKA), have also been validated as primary targets of 4HNE-mediated modification [22], whilst seminal OS has been correlated with altered levels of several proteins mapping to the MAPK/ERK pathways [123]. Such defects have been correlated with a commensurate decrease in global levels of tyrosine phosphorylation in human spermatozoa [22]; a form of post-translational modification that underpins sperm motility, and is particularly important in the induction of hyperactivation [124]. Aside from the targets mentioned above, 4HNE also has the potential to modify sperm phosphatase activity, and hence motility, via the targeting of serine/threonine-protein phosphatase 2B catalytic subunit gamma isoform (PPP3CC) [22]. Indeed, male mice lacking PPP3CC display an infertility phenotype linked to a reduction in sperm motility [125]. Thus, whilst metabolic-related proteins are clearly over represented among those targeted for ROS and lipid aldehyde attack [22], numerous other proteins implicated in sperm motility also appear sensitive to OS.

Recent evidence suggests that mammalian spermatozoa may possess an unfolded protein response (UPR); a conserved cellular pathway that is activated upon accumulation of unfolded or misfolded proteins during stress conditions [126]. The activation of the UPR triggers the expression of chaperones to assist protein refolding (reviewed in [127]), whilst also blocking the synthesis of other proteins via the phosphorylation of eukaryotic translation initiation factor-2 (eIF2α) [128]. Santiago et al. [126] have reported increased levels of proteins involved in the UPR (heat shock proteins; HSF1, HSP90, HSPD1, HSP27; and eIF2α) and reduced motility in human spermatozoa exposed to H_2_O_2_. Despite this, it is possible that prolonged OS within the testes can compromise the UPR owing to 4HNE adduction of key elements of this pathway including the 60 kDa Heat Shock Protein, Mitochondrial (HSPD1) [22]. If this were to be the case, then the inactivation of UPR could contribute to the downregulation of some proteins associated with asthenozoospermia. In any case, such broad-spectrum effects make it extremely challenging to determine whether individual sperm proteins play a dominant role in the pathophysiological responses to OS or whether this phenomenon is instead attributed to dysregulation of multiple targets. This situation is further complicated when considering that 4HNE and other forms of ROS have the potential to damage not only sperm proteins, but also the DNA comprising the paternal and mitochondrial genomes.

### 4.4. Sperm DNA Modifications

At the genomic level, the fidelity of mitochondrial ATP production is controlled by the interplay of mitochondrial (mtDNA) and nuclear (nDNA) DNA, which encode the various components necessary for the proper assembly and function of the mitochondrial complexes of OXPHOS. Specifically, the mtDNA encodes a subset of the protein subunits of Complex I, Complex IV, cytochrome b and ATP synthase, while the nDNA encodes the remainder of the enzymatic components. It follows that the integrity of the mitochondrial and nuclear genomes within the developing germ cell are crucial for the subsequent establishment of normal sperm motility profiles and conversely, that ROS can elicit a deleterious impact on this aspect of sperm function via the induction of DNA damage (reviewed in [129]).

Sperm DNA is highly vulnerable to ROS damage owing to the progressive silencing of the germ cells transcriptional machinery during the latter phases of their development and the attendant reduction in their capacity to mount an effective DNA repair response. Indeed, the replacement of sperm histones with protamines during spermiogenesis promotes extreme compaction of the nDNA; a phenomenon that protects sperm chromatin against ROS-mediated, and other sources, of damage. However, if the integrity of the protamination process is compromised such that portions of poorly compacted DNA remain, these genomic regions are placed at heightened risk of oxidative attack. Accordingly, recent genome wide analyses have demonstrated that oxidative sperm DNA damage occurs predominantly on specific chromatin regions with lower compaction associated with histones and inter-linker domains attached to the nuclear matrix [130,131]. Thus, ROS such as H_2_O_2_, O_2_•^−^ or •OH are all capable of directly damaging DNA integrity by way of base modifications, induction of single- and double-strand DNA brakes, chromatin cross-linking and/or deletions [129]. Additionally, 4HNE has also been shown to promote the formation of DNA adducts such as 8-oxoguanine (8-oxoG), 1,N6-ethenoadenosine and 1,N2-ethenoguanosine in human sperm cells [132]. Accordingly, elevated levels of oxidative DNA damage, and in particular 8-oxoG lesions, are frequently encountered in the spermatozoa of male infertility patients [129,132]; emphasizing the importance of evaluating sperm DNA fragmentation in individuals considering assisted reproduction treatments.

In males with asthenozoospermia, the levels of sperm DNA fragmentation have been found to be significantly higher than that of fertile controls [133]. Bonanno et al. [20] have also reported that asthenozoospermic males had elevated ROS levels in their semen, which was correlated with decreased mtDNA integrity in their spermatozoa. By comparison, nDNA fragmentation was only detected in less than one-fifth of the patients analyzed in this study [20]. This phenomenon may reflect less efficient packaging of mtDNA, rendering it more susceptible to ROS and 4HNE attack [134,135,136]. Alternatively, in spermatozoa the mtDNA is placed in much closer proximity to the main source of ROS generation than that of the nDNA. Aside from changes in mtDNA integrity, it has also been reported that spermatozoa with low-motility possess elevated levels of mtDNA copy number; suggesting that these cells incorporate more mtDNA content during spermatogenesis [20,137,138,139]. By contrast, measurement of cell-free mtDNA copy number in the seminal plasma of asthenozoospermic and oligozoospermic males has identified a reciprocal relationship, whereby lower levels of cell-free mtDNA are associated with increased levels of ROS [21]. A testicular origin for these lesions is supported by the presence of mtDNA deletions in the spermatozoa of infertile males [140,141]. Further, increases in both mtDNA copy number and mtDNA deletions have been recorded in the same patient samples, wherein they were strongly associated with the presence of OS [142].

**Figure 3:**
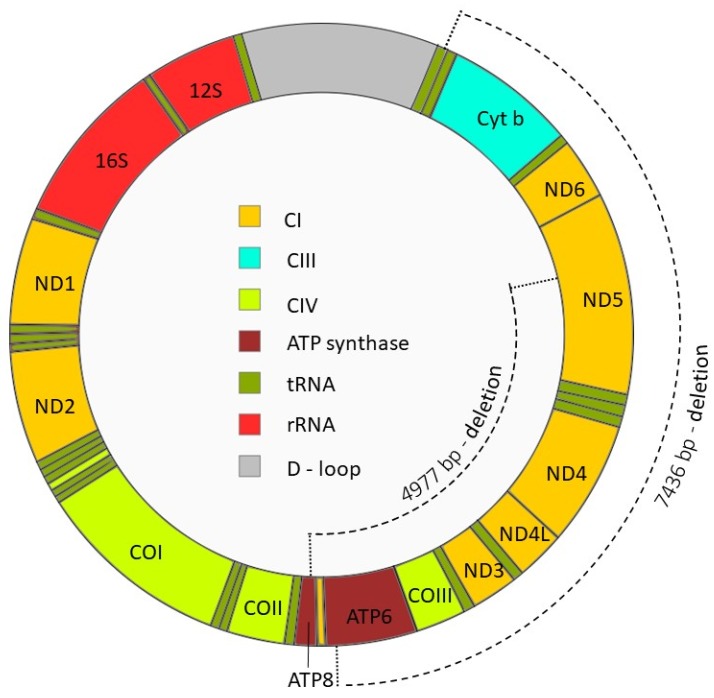
Mitochondrial DNA and location of the mtDNA 4977 bp and 7436 bp deletions. The mtDNA^4977^ deletion includes genes encoding two subunits of ATP-synthase (ATP6 and ATP8), cytochrome oxidase III (COIII), NADH dehydrogenase subunit 3 (ND3), ND4, ND4 subunit L (ND4L), and ND5, whereas mtDNA^7436^ includes ATP6, COIII, ND3, ND4, ND4L, ND5, ND6 and the entire cytochrome b (Cyt b). As a consequence, spermatozoa harboring mtDNA4977 and mtDNA7436 deletions lack several essential OXPHOS genes, fail to assemble functional ETC complexes, and experience compromised energy production. Abbreviations: CI, ETC Complex I; CIII, ETC Complex III; CIV, ETC Complex IV.

One of the best-described large-scale mtDNA deletions is the specific 4977 bp deletion (mtDNA^4977^), which occurs between nucleotides 8470 and 13447 and eliminates seven genes encoding four subunits of Complex I (ND3, ND4, ND4L, partial ND5), one subunit of Complex IV (COIII) and two subunits of ATP-synthase (ATP6 and partial ATP8); all of which are crucial for OXPHOS (depicted in Figure 3). Accordingly, mtDNA^4977^ has been linked to a spectrum of disorders, including heart disease, different forms of cancer and mitochondrial diseases [143,144,145,146]. Notably, this mutation has also been reported to accumulate in different human tissues as a consequence of natural aging [147]. If the mtDNA^4977^ deletion occurs during spermatogenesis and/or spermiogenesis, the mature spermatozoa are endowed with dysfunctional mitochondria, which can act as a source of ROS. Interestingly, mtDNA^4977^ has been recorded in the spermatozoa of infertile males with asthenozoospermia and oligoasthenozoospermia [148,149]. An alternative large-scale mtDNA deletion comprising 7436 bp (mtDNA^7436^) has also been reported in low-motility human spermatozoa [150]. In addition to the genes excised by mtDNA^4977^, mtDNA^7436^ also eliminates genes encoding subunit 6 of Complex I (ND6) and cytochrome b from the mtDNA genome (see Figure 3). Although positive correlations between mtDNA deletions and motility have not been universally established [151,152], Kumar et al. [19] have shown that mutations in sperm mtDNA (giving rise to nucleotide changes in subunits of: ATP6, ATP8, ND2, ND3, ND4 and ND5) do exist in males with oligoasthenozoospermia and that these defects are associated with elevated ROS levels in their spermatozoa. In any case, the crucial role of mitochondria in sperm bioenergetics, as well as the potential for defective mitochondria to generate excessive ROS, motivates a better understanding of the factors responsible for perturbation of mtDNA in developing germ cells.

## 5. Antioxidant Systems in the Male Reproductive Tract

The male reproductive tract produces a wide range of antioxidant scavengers capable of defending spermatozoa from ROS attack. Given that mature spermatozoa are translationally inactive and carry with them minimal endogenous antioxidant defenses, they are highly dependent on these exogenous sources of enzymes during spermatogenesis and their residence in the male reproductive tract. In semen, the most abundant antioxidant enzymes are those belonging to the glutathione peroxidase (GPX) and peroxiredoxin (PRDX) families (reviewed in [153]). The GPX4 isoform is predominantly synthesized in the testis and in mature spermatozoa is abundantly localized in the midpiece. Transgenic mice lacking GPX4 display impaired sperm quality, including deficits in sperm motility and structural abnormalities in the midpiece of the flagellum [154]. An alternative GPX isoform, GPX5, is highly expressed in the caput epididymis and has been implicated in the protection of sperm DNA based on the demonstration that the spermatozoa of *Gpx5*-null mice display lower levels of DNA compaction and higher levels of 8-oxoG than their wildtype counterparts [155]. In addition to GPXs, PRDXs are also highly expressed in the caput and cauda epididymis and have been documented within seminal plasma and in virtually all domains of the human spermatozoon [156]. PRDXs are a highly-conserved family of thiol-dependent peroxidases that regulate antioxidant defense systems by virtue of their ability to reduce H_2_O_2_, peroxynitrite (ONOO^−^) and hydroperoxides (ROOH); themselves becoming inactivated upon oxidization [156,157]. PRDX1 and PRDX6 have been shown to be abundantly expressed in rat epididymal spermatozoa and to become highly oxidized after the induction of OS with substrates such as tert-butyl hydroperoxide (tert-BHP) [158]. Due to its catalase and calcium-independent phospholipase A2 (Ca^2+^-iPLA2) activities, PRDX6 has also been implicated in the prevention of LPO and the repair of oxidized membranes [159]. Accordingly, the inhibition of PRDX6 Ca^2+^-iPLA2 activity in human spermatozoa has recently been shown to promote extensive oxidative damage (including high levels of LPO, DNA oxidation and reduced ∆Ѱ), which contributes to a phenotype of reduced sperm motility [160]. Aside from GPXs and PRDXs, alternative enzymatic antioxidants such as superoxide dismutase (SOD) and catalase (CAT) collaborate to protect spermatozoa held within male reproductive tract. Such enzymatic defenses can be supplemented by dietary derived non-enzymatic antioxidants, including vitamins (C, E and B), carotenoids, glutathione, inositol, carnitines, cysteines, hyaluronan, serum albumin, and zinc (reviewed in [161]).

Highlighting the physiological importance of these collective antioxidant defenses, patients with poor sperm motility parameters commonly have an attendant deficit in their levels of seminal plasma antioxidants. By way of example, studies of asthenozoospermic individuals with idiopathic or varicocele-related background have shown these lesions are commonly accompanied by relatively low levels of PRDX6 and PRDX1 enzymes within their seminal plasma and spermatozoa, respectively [162]. Similarly, the levels of lactoferrin (LTF; an iron-binding glycoprotein secreted by the mammalian epididymis [163,164] that possesses antioxidant properties and is capable of binding receptors on the sperm head and midpiece [165]) are reduced in spermatozoa from asthenozoospermic males [166]. Whilst others have reported the opposing trend; i.e., higher levels of LTF in spermatozoa from males with asthenozoospermia [18], such discrepancies may reflect the alternative defects that give rise to this pathology and/or the integrity of sperm membrane domains wherein LTF receptors are localized. Building on this body of evidence, the levels of vitamin C, vitamin E and the reduced form of glutathione (GSH; an endogenous antioxidant that can function synergistically with vitamin C [167]), have each been found to be diminished in the semen of asthenozoospermic males compared to normozoospermic controls [168,169]. In contrast to this general trend, data from meta-analyses have failed to document significant changes in the levels of alternative seminal plasma antioxidants such as SOD between males with different forms of infertility (including oligoasthenozoospermia) and that of healthy controls [170], suggesting that not all antioxidants are of equivalent importance in terms of maintaining sperm function. One possible explanation is that the attenuation of antioxidant capacity in infertile individuals may be due, at least in part, to redox driven modifications of specific antioxidant enzymes. Illustrative of this, PRDXs, which serve as primary antioxidants in ejaculated human spermatozoa, are especially vulnerable to ROS-induced modifications such as S-nitrosylation, tyrosine nitration, and S-glutathionylation (reviewed in [171]). Oxidative damage to specific antioxidants may therefore be among the first steps in the cascade of events that contribute to OS-mediated male infertility.

Taking into account the potential of antioxidants to ameliorate OS related pathologies, it is perhaps not surprising that different cocktails of enzymatic and non-enzymatic antioxidants have found therapeutic application during assisted reproductive interventions [172]. Among the most popular of these are vitamin C, zinc and L-carnitine [173,174,175], although it must be acknowledged the outcomes of these trials are far from consistent. In this context, zinc supplementation has been reported to improve sperm motility by way of reducing OS, apoptosis and DNA fragmentation; but notably, these results were only achieved in the presence of vitamins C and E [174]. Similarly, in a study by Garolla et al. [175], L-carnitine was shown to improve sperm motility but only in samples wherein normal levels of GPX were maintained. This caveat could explain the opposing data obtained by Sigman et al. [176], who reported that L-carnitine had no effect on sperm motility. In view of these dichotomous results, there is a clear imperative to continue basic research to advance our understanding of the interplay of ROS and sperm biology in order to inform the development of effective therapies to combat OS-mediated lesions.

## 6. Conclusions

Overall, this review highlights mechanisms contributing to an OS-induced decline in sperm motility associated with conditions such as asthenozoospermia. A clear consensus to emerge from the reviewed literature is that the presence of OS in the male reproductive tract is strongly and positively correlated with reduced sperm motility. This state of OS can be evoked not only by intrinsic factors but also by a diversity of environmental agents commonly encountered during modern life. Thus, the challenges presented to the male reproductive tract in terms of mounting an effective and prolonged defense against ROS can result in an attenuation of antioxidant capacity and a concomitant acceleration of OS-induced sperm damage. One particular pathway that appears to contribute to much of the OS response is that of LPO, which is responsible for the generation of highly reactive aldehyde species. These electrophiles are able to adversely impact sperm motility via the adduction and dysregulation of proteins involved in sperm bioenergetic pathways as well as the structural and signaling components of the motility apparatus. Given that these lesions go hand in hand with oxidative DNA damage, it is possible that they may serve a physiological role in terms of reducing the likelihood of such sperm from participating in fertilization and thus transmitting an altered paternal genome to the next generation. This possibility emphasizes the need for the development of novel therapeutic interventions to address the burden of OS-mediated dysfunction in the male germline.

## References

[1] Brugh V.M., Lipshultz L.I. (2004). Male factor infertility: Evaluation and management. Clin. North Am..

[2] Lotti F., Maggi M. (2015). Ultrasound of the male genital tract in relation to male reproductive health. Hum. Reprod. Update.

[3] Vergallo A., Giampietri L., Baldacci F., Volpi L., Chico L., Pagni C., Giorgi F.S., Ceravolo R., Tognoni G., Siciliano G. (2018). Oxidative Stress Assessment in Alzheimer’s Disease: A Clinic Setting Study. Am. J. Alzheimers Dis. Other Demen..

[4] Ma-On C., Sanpavat A., Whongsiri P., Suwannasin S., Hirankarn N., Tangkijvanich P., Boonla C. (2017). Oxidative stress indicated by elevated expression of Nrf2 and 8-OHdG promotes hepatocellular carcinoma progression. Med. Oncol..

[5] Li B., Chi R.F., Qin F.Z., Guo X.F. (2016). Distinct changes of myocyte autophagy during myocardial hypertrophy and heart failure: Association with oxidative stress. Exp. Physiol..

[6] Dursun E., Akalin F.A., Genc T., Cinar N., Erel O., Yildiz B.O. (2016). Oxidative Stress and Periodontal Disease in Obesity. Medicine (Baltimore).

[7] Lanzafame F.M., La Vignera S., Vicari E., Calogero A.E. (2009). Oxidative stress and medical antioxidant treatment in male infertility. Reprod. Biomed. Online.

[8] Agarwal A., Prabakaran S., Allamaneni S.S. (2006). Relationship between oxidative stress, varicocele and infertility: A meta-analysis. Reprod. Biomed. Online.

[9] Fraczek M., Sanocka D., Kamieniczna M., Kurpisz M. (2008). Proinflammatory cytokines as an intermediate factor enhancing lipid sperm membrane peroxidation in in vitro conditions. J. Androl..

[10] Kurfurstova D., Bartkova J., Vrtel R., Mickova A., Burdova A., Majera D., Mistrik M., Kral M., Santer F.R., Bouchal J. (2016). DNA damage signalling barrier, oxidative stress and treatment-relevant DNA repair factor alterations during progression of human prostate cancer. Mol. Oncol..

[11] Walters J.L.H., De Iuliis G.N., Nixon B., Bromfield E.G. (2018). Oxidative Stress in the Male Germline: A Review of Novel Strategies to Reduce 4-Hydroxynonenal Production. Antioxidants.

[12] Agarwal A., Mulgund A., Sharma R., Sabanegh E. (2014). Mechanisms of oligozoospermia: An oxidative stress perspective. Syst. Biol. Reprod. Med..

[13] Muratori M., Tamburrino L., Marchiani S., Cambi M., Olivito B., Azzari C., Forti G., Baldi E. (2015). Investigation on the Origin of Sperm DNA Fragmentation: Role of Apoptosis, Immaturity and Oxidative Stress. Mol. Med..

[14] Koppers A.J., Mitchell L.A., Wang P., Lin M., Aitken R.J. (2011). Phosphoinositide 3-kinase signalling pathway involvement in a truncated apoptotic cascade associated with motility loss and oxidative DNA damage in human spermatozoa. Biochem J..

[15] Aitken R.J., Smith T.B., Jobling M.S., Baker M.A., De Iuliis G.N. (2014). Oxidative stress and male reproductive health. Asian J. Androl..

[16] World Health Organization, Department of Reproductive Health and Research (2010). WHO Laboratory Manual for the Examination and Processing of Human Semen.

[17] Liu F.J., Liu X., Han J.L., Wang Y.W., Jin S.H., Liu X.X., Liu J., Wang W.T., Wang W.J. (2015). Aged men share the sperm protein PATE1 defect with young asthenozoospermia patients. Hum. Reprod..

[18] Nowicka-Bauer K., Lepczynski A., Ozgo M., Kamieniczna M., Fraczek M., Stanski L., Olszewska M., Malcher A., Skrzypczak W., Kurpisz M.K. (2018). Sperm mitochondrial dysfunction and oxidative stress as possible reasons for isolated asthenozoospermia. J. Physiol. Pharmacol..

[19] Kumar R., Venkatesh S., Kumar M., Tanwar M., Shasmsi M.B., Kumar R., Gupta N.P., Sharma R.K., Talwar P., Dada R. (2009). Oxidative stress and sperm mitochondrial DNA mutation in idiopathic oligoasthenozoospermic men. Indian J. Biochem. Biophys..

[20] Bonanno O., Romeo G., Asero P., Pezzino F.M., Castiglione R., Burrello N., Sidoti G., Frajese G.V., Vicari E., D’Agata R. (2016). Sperm of patients with severe asthenozoospermia show biochemical, molecular and genomic alterations. Reproduction.

[21] Chen Y., Liao T., Zhu L., Lin X., Wu R., Jin L. (2018). Seminal plasma cell-free mitochondrial DNA copy number is associated with human semen quality. Eur. J. Obstet. Gynecol. Reprod. Biol..

[22] Baker M.A., Weinberg A., Hetherington L., Villaverde A.I., Velkov T., Baell J., Gordon C.P. (2015). Defining the mechanisms by which the reactive oxygen species by-product, 4-hydroxynonenal, affects human sperm cell function. Biol. Reprod..

[23] Gavella M., Lipovac V. (1992). NADH-dependent oxidoreductase (diaphorase) activity and isozyme pattern of sperm in infertile men. Arch. Androl..

[24] Jastroch M., Divakaruni A.S., Mookerjee S., Treberg J.R., Brand M.D. (2010). Mitochondrial proton and electron leaks. Essays Biochem..

[25] O’Flaherty C.M., Beorlegui N.B., Beconi M.T. (1999). Reactive oxygen species requirements for bovine sperm capacitation and acrosome reaction. Theriogenology.

[26] Koppenol W.H. (2001). The Haber-Weiss cycle—70 years later. Redox Rep..

[27] Buzadzic B., Vucetic M., Jankovic A., Stancic A., Korac A., Korac B., Otasevic V. (2015). New insights into male (in)fertility: The importance of NO. Br. J. Pharmacol..

[28] Otasevic V., Stancic A., Korac A., Jankovic A., Korac B. (2019). Reactive oxygen, nitrogen, and sulfur species in human male fertility. A crossroad of cellular signaling and pathology. Biofactors.

[29] Chen S.J., Allam J.P., Duan Y.G., Haidl G. (2013). Influence of reactive oxygen species on human sperm functions and fertilizing capacity including therapeutical approaches. Arch. Gynecol. Obstet..

[30] Alomar M., Alzoabi M., Zarkawi M. (2016). Kinetics of hydrogen peroxide generated from live and dead ram spermatozoa and the effects of catalase and oxidase substrates addition. Czech, J. Anim. Sci..

[31] Gao W., Pu Y., Luo K.Q., Chang D.C. (2001). Temporal relationship between cytochrome c release and mitochondrial swelling during UV-induced apoptosis in living HeLa cells. J. Cell Sci..

[32] Patil P.S., Humbarwadi R.S., Patil A.D., Gune A.R. (2013). Immature germ cells in semen-correlation with total sperm count and sperm motility. J. Cytol..

[33] Hermo L., Pelletier R.M., Cyr D.G., Smith C.E. (2010). Surfing the wave, cycle, life history, and genes/proteins expressed by testicular germ cells. Part 1: Background to spermatogenesis, spermatogonia, and spermatocytes. Microsc. Res. Tech..

[34] Gomez E., Buckingham D.W., Brindle J., Lanzafame F., Irvine D.S., Aitken R.J. (1996). Development of an image analysis system to monitor the retention of residual cytoplasm by human spermatozoa: Correlation with biochemical markers of the cytoplasmic space, oxidative stress, and sperm function. J. Androl..

[35] Agarwal A., Rana M., Qiu E., AlBunni H., Bui A.D., Henkel R. (2018). Role of oxidative stress, infection and inflammation in male infertility. Andrologia.

[36] Plante M., de Lamirande E., Gagnon C. (1994). Reactive oxygen species released by activated neutrophils, but not by deficient spermatozoa, are sufficient to affect normal sperm motility. Fertil. Steril..

[37] Wang A., Fanning L., Anderson D.J., Loughlin K.R. (1997). Generation of reactive oxygen species by leukocytes and sperm following exposure to urogenital tract infection. Arch. Androl..

[38] Tamura M., Tamura T., Tyagi S.R., Lambeth J.D. (1988). The superoxide-generating respiratory burst oxidase of human neutrophil plasma membrane. Phosphatidylserine as an effector of the activated enzyme. J. Biol. Chem..

[39] Bostwick D.G., Ma J. (2020). Spermatic Cord and Testicular Adnexa. Urologic Surgical Pathology.

[40] Practice Committee of the American Society for Reproductive Medicine, Society for Male Reproduction and Urology (2014). Report on varicocele and infertility: A committee opinion. Fertil. Steril..

[41] Hendin B.N., Kolettis P.N., Sharma R.K., Thomas A.J., Agarwal A. (1999). Varicocele is associated with elevated spermatozoal reactive oxygen species production and diminished seminal plasma antioxidant capacity. J. Urol..

[42] Sakamoto Y., Ishikawa T., Kondo Y., Yamaguchi K., Fujisawa M. (2008). The assessment of oxidative stress in infertile patients with varicocele. BJU Int..

[43] Agarwal A., Hamada A., Esteves S.C. (2012). Insight into oxidative stress in varicocele-associated male infertility: Part 1. Nat. Rev. Urol..

[44] Mostafa T., Rashed L., Taymour M. (2016). Seminal cyclooxygenase relationship with oxidative stress in infertile oligoasthenoteratozoospermic men with varicocele. Andrologia.

[45] Gul M., Bugday M.S., Erel O. (2018). Thiol-disulphide homoeostasis as an oxidative stress marker in men with varicocele. Andrologia.

[46] Erfani Majd N., Sadeghi N., Tavalaee M., Tabandeh M.R., Nasr-Esfahani M.H. (2019). Evaluation of Oxidative Stress in Testis and Sperm of Rat Following Induced Varicocele. Urol. J..

[47] Cho C.L., Esteves S.C., Agarwal A. (2016). Novel insights into the pathophysiology of varicocele and its association with reactive oxygen species and sperm DNA fragmentation. Asian J. Androl..

[48] Bedford J.M. (2015). Human spermatozoa and temperature: The elephant in the room. Biol. Reprod..

[49] Ikeda M., Kodama H., Fukuda J., Shimizu Y., Murata M., Kumagai J., Tanaka T. (1999). Role of radical oxygen species in rat testicular germ cell apoptosis induced by heat stress. Biol. Reprod..

[50] Paul C., Murray A.A., Spears N., Saunders P.T. (2008). A single, mild, transient scrotal heat stress causes DNA damage, subfertility and impairs formation of blastocysts in mice. Reproduction.

[51] Saikhun J., Kitiyanant Y., Vanadurongwan V., Pavasuthipaisit K. (1998). Effects of sauna on sperm movement characteristics of normal men measured by computer-assisted sperm analysis. Int. J. Androl..

[52] Shefi S., Tarapore P.E., Walsh T.J., Croughan M., Turek P.J. (2007). Wet heat exposure: A potentially reversible cause of low semen quality in infertile men. Int. Braz. J. Urol..

[53] Garolla A., Torino M., Sartini B., Cosci I., Patassini C., Carraro U., Foresta C. (2013). Seminal and molecular evidence that sauna exposure affects human spermatogenesis. Hum. Reprod..

[54] Houston B.J., Nixon B., Martin J.H., De Iuliis G.N., Trigg N.A., Bromfield E.G., McEwan K.E., Aitken R.J. (2018). Heat exposure induces oxidative stress and DNA damage in the male germ line. Biol. Reprod..

[55] Houston B.J., Nixon B., King B.V., De Iuliis G.N., Aitken R.J. (2016). The effects of radiofrequency electromagnetic radiation on sperm function. Reproduction.

[56] Agarwal A., Singh A., Hamada A., Kesari K. (2011). Cell phones and male infertility: A review of recent innovations in technology and consequences. Int. Braz. J. Urol..

[57] Wdowiak A., Mazurek P.A., Wdowiak A., Bojar I. (2017). Effect of electromagnetic waves on human reproduction. Ann. Agric. Environ. Med..

[58] Koppers A.J., De Iuliis G.N., Finnie J.M., McLaughlin E.A., Aitken R.J. (2008). Significance of mitochondrial reactive oxygen species in the generation of oxidative stress in spermatozoa. J. Clin. Endocrinol. Metab..

[59] De Iuliis G.N., Newey R.J., King B.V., Aitken R.J. (2009). Mobile phone radiation induces reactive oxygen species production and DNA damage in human spermatozoa in vitro. PLoS ONE.

[60] Feng B., Qiu L., Ye C., Chen L., Fu Y., Sun W. (2016). Exposure to a 50-Hz magnetic field induced mitochondrial permeability transition through the ROS/GSK-3β signaling pathway. Int. J. Radiat. Biol..

[61] Wijesekara G.U., Fernando D.M., Wijerathna S., Bandara N. (2015). Environmental and occupational exposures as a cause of male infertility. Ceylon Med. J..

[62] Saad A.B., Rjeibi I., Alimi H., Ncib S., Smida A., Zouari N., Zourgui L. (2017). Lithium induced, oxidative stress and related damages in testes and heart in male rats: The protective effects of Malva sylvestris extract. Biomed. Pharmacother..

[63] Kaur S., Saluja M., Bansal M.P. (2018). Bisphenol A induced oxidative stress and apoptosis in mice testes: Modulation by selenium. Andrologia.

[64] Du J., Xiong D., Zhang Q., Li X., Liu X., You H., Ding S., Yang X., Yuan J. (2017). Mono-butyl phthalate-induced mouse testis injury is associated with oxidative stress and down-regulated expression of Sox9 and *Dazl*. J. Toxicol. Sci..

[65] Wang Y.X., Zeng Q., Sun Y., You L., Wang P., Li M., Yang P., Li J., Huang Z., Wang C. (2016). Phthalate exposure in association with serum hormone levels, sperm DNA damage and spermatozoa apoptosis: A cross-sectional study in China. Environ. Res..

[66] Saleh R.A., Agarwal A., Sharma R.K., Nelson D.R., Thomas A.J. (2002). Effect of cigarette smoking on levels of seminal oxidative stress in infertile men: A prospective study. Fertil. Steril..

[67] Hamad M.F., Shelko N., Kartarius S., Montenarh M., Hammadeh M.E. (2014). Impact of cigarette smoking on histone (H2B) to protamine ratio in human spermatozoa and its relation to sperm parameters. Andrology.

[68] Oh S.I., Lee M.S., Kim C.I., Song K.Y., Park S.C. (2002). Aspartate modulates the ethanol-induced oxidative stress and glutathione utilizing enzymes in rat testes. Exp. Mol. Med..

[69] Siervo G.E., Vieira H.R., Ogo F.M., Fernandez C.D., Gonçalves G.D., Mesquita S.F., Anselmo-Franci J.A., Cecchini R., Guarnier F.A., Fernandes G.S. (2015). Spermatic and testicular damages in rats exposed to ethanol: Influence of lipid peroxidation but not testosterone. Toxicology.

[70] Aitken R.J., Baker M.A., De Iuliis G.N., Nixon B., Habenicht U.F., Aitken R. (2010). New Insights into Sperm Physiology and Pathology. Fertility Control. Handbook of Experimental Pharmacology.

[71] Lenzi A., Gandini L., Picardo M., Tramer F., Sandri G., Panfili E. (2000). Lipoperoxidation damage of spermatozoa polyunsaturated fatty acids (PUFA): Scavenger mechanisms and possible scavenger therapies. Front. Biosci..

[72] Hauck A.K., Bernlohr D.A. (2016). Oxidative stress and lipotoxicity. J. Lipid Res..

[73] Bromfield E.G., Aitken R.J., McLaughlin E.A., Nixon B. (2017). Proteolytic degradation of heat shock protein A2 occurs in response to oxidative stress in male germ cells of the mouse. Mol. Hum. Reprod..

[74] Badouard C., Ménézo Y., Panteix G., Ravanat J.L., Douki T., Cadet J., Favier A. (2008). Determination of new types of DNA lesions in human sperm. Zygote.

[75] Dalleau S., Baradat M., Guéraud F., Huc L. (2013). Cell death and diseases related to oxidative stress: 4-hydroxynonenal (HNE) in the balance. Cell Death Differ..

[76] LoPachin R.M., Gavin T., Petersen D.R., Barber D.S. (2009). Molecular mechanisms of 4-hydroxy-2-nonenal and acrolein toxicity: Nucleophilic targets and adduct formation. Chem. Res. Toxicol..

[77] Schaur R.J., Siems W., Bresgen N., Eckl P.M. (2015). 4-Hydroxy-nonenal-A Bioactive Lipid Peroxidation Product. Biomolecules.

[78] Doorn J.A., Petersen D.R. (2003). Covalent adduction of nucleophilic amino acids by 4-hydroxynonenal and 4-oxononenal. Chem. Biol. Interact..

[79] Bromfield E.G., Aitken R.J., Anderson A.L., McLaughlin E.A., Nixon B. (2015). The impact of oxidative stress on chaperone-mediated human sperm-egg interaction. Hum. Reprod..

[80] Morielli T., O’Flaherty C. (2015). Oxidative stress impairs function and increases redox protein modifications in human spermatozoa. Reproduction.

[81] Salvolini E., Buldreghini E., Lucarini G., Vignini A., Di Primio R., Balercia G. (2012). Nitric oxide synthase and tyrosine nitration in idiopathic asthenozoospermia: An immunohistochemical study. Fertil. Steril..

[82] Vignini A., Nanetti L., Buldreghini E., Moroni C., Ricciardo-Lamonica G., Mantero F., Boscaro M., Mazzanti L., Balercia G. (2006). The production of peroxynitrite by human spermatozoa may affect sperm motility through the formation of protein nitrotyrosine. Fertil. Steril..

[83] Ramirez J.P., Carreras A., Mendoza C. (1992). Sperm plasma membrane integrity in fertile and infertile men. Andrologia.

[84] Karimfar M.H., Niazvand F., Haghani K., Ghafourian S., Shirazi R., Bakhtiyari S. (2015). The protective effects of melatonin against cryopreservation-induced oxidative stress in human sperm. Int. J. Immunopathol. Pharmacol..

[85] Ghorbani M., Vatannejad A., Khodadadi I., Amiri I., Tavilani H. (2016). Protective effects of glutathione supplementation against oxidative stress during cryopreservation of human spermatozoa. Cryo. Letters.

[86] Najafi A., Daghigh Kia H., Mehdipour M., Shamsollahi M., Miller D.J. (2019). Does fennel extract ameliorate oxidative stress frozen-thawed ram sperm?. Cryobiology.

[87] Varela E., Rojas M., Restrepo G. (2019). Membrane stability and mitochondrial activity of bovine sperm frozen with low-density lipoproteins and trehalose. Reprod. Domest. Anim..

[88] Dashtestani F., Ghourchian H., Najafi A. (2019). Silver-gold-apoferritin nanozyme for suppressing oxidative stress during cryopreservation. Mater. Sci. Eng. C. Mater. Biol. Appl..

[89] Mishra A.K., Kumar A., Swain D.K., Yadav S., Nigam R. (2018). Insights into pH regulatory mechanisms in mediating spermatozoa functions. Vet. World.

[90] Elinder F., Liin S.I. (2017). Actions and Mechanisms of Polyunsaturated Fatty Acids on Voltage-Gated Ion Channels. Front. Physiol..

[91] Hosseinzadeh Colagar A., Karimi F., Jorsaraei S.G. (2013). Correlation of sperm parameters with semen lipid peroxidation and total antioxidants levels in astheno- and oligoasheno- teratospermic men. Iran. Red. Crescent. Med. J..

[92] Tamburrino L., Marchiani S., Vicini E., Muciaccia B., Cambi M., Pellegrini S., Forti G., Muratori M., Baldi E. (2015). Quantification of CatSper1 expression in human spermatozoa and relation to functional parameters. Hum. Reprod..

[93] Liu S.W., Li Y., Zou L.L., Guan Y.T., Peng S., Zheng L.X., Deng S.M., Zhu L.Y., Wang L.W., Chen L.X. (2017). Chloride channels are involved in sperm motility and are downregulated in spermatozoa from patients with asthenozoospermia. Asian J. Androl..

[94] Sinha A., Singh V., Singh S., Yadav S. (2019). Proteomic analyses reveal lower expression of TEX40 and ATP6V0A2 proteins related to calcium ion entry and acrosomal acidification in asthenozoospermic males. Life Sci..

[95] Hashemitabar M., Sabbagh S., Orazizadeh M., Ghadiri A., Bahmanzadeh M. (2015). A proteomic analysis on human sperm tail: Comparison between normozoospermia and asthenozoospermia. J. Assist. Reprod. Genet..

[96] Agarwal A., Sharma R., Durairajanayagam D., Ayaz A., Cui Z., Willard B., Gopalan B., Sabanegh E. (2015). Major protein alterations in spermatozoa from infertile men with unilateral varicocele. Reprod. Biol. Endocrinol..

[97] Ursini F., Heim S., Kiess M., Maiorino M., Roveri A., Wissing J., Flohe L. (1999). Dual function of the selenoprotein PHGPx during sperm maturation. Science.

[98] Bahr G.F., Engler W.F. (1970). Considerations of volume, mass, DNA, and arrangement of mitochondria in the midpiece of bull spermatozoa. Exp. Cell Res..

[99] Parker N., Vidal-Puig A., Brand M.D. (2008). Stimulation of mitochondrial proton conductance by hydroxynonenal requires a high membrane potential. Biosci. Rep..

[100] Hanukoglu I., Rapoport R., Weiner L., Sklan D. (1993). Electron leakage from the mitochondrial NADPH–adrenodoxin reductase–adrenodoxin–P450scc (cholesterol side chain cleavage) system. Arch. Biochem. Biophys..

[101] Amaral A., Ramalho-Santos J. (2010). Assessment of mitochondrial potential: Implications for the correct monitoring of human sperm function. Int. J. Androl..

[102] Zhang W.D., Zhang Z., Jia L.T., Zhang L.L., Fu T., Li Y.S., Wang P., Sun L., Shi Y., Zhang H.Z. (2014). Oxygen free radicals and mitochondrial signaling in oligospermia and asthenospermia. Mol. Med. Rep..

[103] Amaral A., Castillo J., Estanyol J.M., Ballesca J.L., Ramalho-Santos J., Oliva R. (2013). Human sperm tail proteome suggests new endogenous metabolic pathways. Mol. Cell Proteom..

[104] Amaral A., Castillo J., Ramalho-Santos J., Oliva R. (2014). The combined human sperm proteome: Cellular pathways and implications for basic and clinical science. Hu. Reprod. Update.

[105] Moscatelli N., Lunetti P., Braccia C., Armirotti A., Pisanello F., De Vittorio M., Zara V., Ferramosca A. (2019). Comparative Proteomic Analysis of Proteins Involved in Bioenergetics Pathways Associated with Human Sperm Motility. Int. J. Mol. Sci..

[106] Aitken R.J., Whiting S., De Iuliis G.N., McClymont S., Mitchell L.A., Baker M.A. (2012). Electrophilic aldehydes generated by sperm metabolism activate mitochondrial reactive oxygen species generation and apoptosis by targeting succinate dehydrogenase. J. Biol. Chem..

[107] Carbone D.L., Doorn J.A., Kiebler Z., Sampey B.P., Petersen D.R. (2004). Inhibition of Hsp72-mediated protein refolding by 4-hydroxy-2-nonenal. Chem. Res. Toxicol..

[108] Carbone D.L., Doorn J.A., Petersen D.R. (2004). 4-Hydroxynonenal regulates 26S proteasomal degradation of alcohol dehydrogenase. Free Radic. Biol. Med..

[109] Amaral A., Paiva C., Attardo Parrinello C., Estanyol J.M., Ballescà J.L., Ramalho-Santos J., Oliva R. (2014). Identification of proteins involved in human sperm motility using highthroughput differential proteomics. J. Proteome Res..

[110] Guo Y., Jiang W., Yu W., Niu X., Liu F., Zhou T., Zhang H., Li Y., Zhu H., Zhou Z. (2019). Proteomics analysis of asthenozoospermia and identification of glucose-6-phosphate isomerase as an important enzyme for sperm motility. J. Proteom..

[111] Miki K., Qu W., Goulding E.H., Willis W.D., Bunch D.O., Strader L.F., Perreault S.D., Eddy E.M., O’Brien D.A. (2004). Glyceraldehyde 3-phosphate dehydrogenase-S, a sperm-specific glycolytic enzyme, is required for sperm motility and male fertility. Proc. Natl. Acad. Sci. USA.

[112] Elkina Y.L., Atroshchenko M.M., Bragina E.E., Muronetz V.I., Schmalhausen E.V. (2011). Oxidation of glyceraldehyde-3-phosphate dehydrogenase decreases sperm motility. Biochemistry.

[113] Liu J., Wang Y., Gong L., Sun C. (2015). Oxidation of glyceraldehyde-3-phosphate dehydrogenase decreases sperm motility in diabetes mellitus. Biochem. Biophys. Res. Commun..

[114] Yang Y., Cheng L., Wang Y., Han Y., Liu J., Deng X., Chao L. (2017). Expression of NDUFA13 in asthenozoospermia and possible pathogenesis. Reprod. Biomed. Online.

[115] Roberts A.J., Kon T., Knight P.J., Sutoh K., Burgess S.A. (2013). Functions and mechanics of dynein motor proteins. Nat. Rev. Mol. Cell Biol..

[116] Brokaw C.J. (1972). Flagellar movement: A sliding filament model. Science.

[117] Zhao W., Li Z., Ping P., Wang G., Yuan X., Sun F. (2018). Outer dense fibers stabilize the axoneme to maintain sperm motility. J. Cell Mol. Med..

[118] Linck R.W., Chemes H., Albertini D.F. (2016). The axoneme: The propulsive engine of spermatozoa and cilia and associated ciliopathies leading to infertility. J. Assist. Reprod. Genet..

[119] Eddy E.M., Toshimori K., O’Brien D.A. (2003). Fibrous sheath of mammalian spermatozoa. Microsc. Res. Tech..

[120] Brown P.R., Miki K., Harper D.B., Eddy E.M. (2003). A-kinase anchoring protein 4 binding proteins in the fibrous sheath of the sperm flagellum. Biol. Reprod..

[121] Nixon B., Bernstein I., Cafe S.L., Delehedde M., Sergeant N., Eamens A.L., Lord T., Dun M.D., De Iuliis G.N., Bromfield E.G. (2019). A Kinase Anchor Protein 4 is vulnerable to oxidative adduction in male germ cells. Front. Cell Dev. Biol..

[122] Miki K., Willis W.D., Brown P.R., Goulding E.H., Fulcher K.D., Eddy E.M. (2002). Targeted disruption of the Akap4 gene causes defects in sperm flagellum and motility. Dev. Biol..

[123] Ayaz A., Agarwal A., Sharma R., Kothandaraman N., Cakar Z., Sikka S. (2018). Proteomic analysis of sperm proteins in infertile men with high levels of reactive oxygen species. Andrologia.

[124] Nassar A., Mahony M., Morshedi M., Lin M.H., Srisombut C., Oehninger S. (1999). Modulation of sperm tail protein tyrosine phosphorylation by pentoxifylline and its correlation with hyperactivated motility. Fertil. Steril..

[125] Miyata H., Satouh Y., Mashiko D., Muto M., Nozawa K., Shiba K., Fujihara Y., Isotani A., Inaba K., Ikawa M. (2015). Sperm calcineurin inhibition prevents mouse fertility with implications for male contraceptive. Science.

[126] Santiago J., Silva J.V., Fardilha M. (2019). First Insights on the Presence of the Unfolded Protein Response in Human Spermatozoa. Int. J. Mol. Sci..

[127] Jovaisaite V., Mouchiroud L., Auwerx J. (2014). The mitochondrial unfolded protein response, a conserved stress response pathway with implications in health and disease. J. Exp. Biol..

[128] Zhang G., Ling X., Liu K., Wang Z., Zou P., Gao J., Cao J., Ao L. (2016). The p-eIF2α/ATF4 pathway links endoplasmic reticulum stress to autophagy following the production of reactive oxygen species in mouse spermatocyte-derived cells exposed to dibutyl phthalate. Free Radic. Res..

[129] Cocuzza M., Sikka S.C., Athayde K.S., Agarwal A. (2007). Clinical relevance of oxidative stress and sperm chromatin damage in male infertility: An evidence based analysis. Int. Braz. J. Urol..

[130] Xavier M.J., Nixon B., Roman S.D., Scott R.J., Drevet J.R., Aitken R.J. (2019). Paternal impacts on development: Identification of genomic regions vulnerable to oxidative DNA damage in human spermatozoa. Hum. Reprod..

[131] Xavier M.J., Nixon B., Roman S.D., Aitken R.J. (2018). Improved methods of DNA extraction from human spermatozoa that mitigate experimentally-induced oxidative DNA damage. PLoS ONE.

[132] Kodama H., Yamaguchi R., Fukuda J., Kasai H., Tanaka T. (1997). Increased oxidative deoxyribonucleic acid damage in the spermatozoa of infertile male patients. Fertil. Steril..

[133] Piasecka M., Gaczarzewicz D., Laszczyńska M., Starczewski A., Brodowska A. (2007). Flow cytometry application in the assessment of sperm DNA integrity of men with asthenozoospermia. Folia Histochem. Cytobiol..

[134] Yakes F.M., Van Houten B. (1997). Mitochondrial DNA damage is more extensive and persists longer than nuclear DNA damage in human cells following oxidative stress. Proc. Natl. Acad. Sci. USA.

[135] Salazar J.J., Van Houten B. (1997). Preferential mitochondrial DNA injury caused by glucose oxidase as a steady generator of hydrogen peroxide in human fibroblasts. Mutat. Res..

[136] Sawyer D.E., Mercer B.G., Wiklendt A.M., Aitken R.J. (2003). Quantitative analysis of gene-specific DNA damage in human spermatozoa. Mutat. Res..

[137] Díez-Sánchez C., Ruiz-Pesini E., Lapeña A.C., Montoya J., Pérez-Martos A., Enríquez J.A., López-Pérez M.J. (2003). Mitochondrial DNA content of human spermatozoa. Biol. Reprod..

[138] Amaral A., Ramalho-Santos J., St John J.C. (2007). The expression of polymerase gamma and mitochondrial transcription factor A and the regulation of mitochondrial DNA content in mature human sperm. Hum. Reprod..

[139] Song G.J., Lewis V. (2008). Mitochondrial DNA integrity and copy number in sperm from infertile men. Fertil. Steril..

[140] Kao S., Chao H.T., Wei Y.H. (1995). Mitochondrial deoxyribonucleic acid 4977-bp deletion is associated with diminished fertility and motility of human sperm. Biol. Reprod..

[141] Gashti N.G., Salehi Z., Madani A.H., Dalivandan S.T. (2014). 4977-bp mitochondrial DNA deletion in infertile patients with varicocele. Andrologia.

[142] Lin P.H., Lee S.H., Su C.P., Wei Y.H. (2003). Oxidative damage to mitochondrial DNA in atrial muscle of patients with atrial fibrillation. Free Radic. Biol. Med..

[143] Vecoli C., Borghini A., Pulignani S., Mercuri A., Turchi S., Carpeggiani C., Picano E., Andreassi M.G. (2018). Prognostic value of mitochondrial DNA4977 deletion and mitochondrial DNA copy number in patients with stable coronary artery disease. Atherosclerosis.

[144] Dimberg J., Hong T.T., Nguyen L.T.T., Skarstedt M., Löfgren S., Matussek A. (2015). Common 4977 bp deletion and novel alterations in mitochondrial DNA in Vietnamese patients with breast cancer. Springerplus.

[145] Guo Z.S., Jin C.L., Yao Z.J., Wang Y.M., Xu B.T. (2017). Analysis of the Mitochondrial 4977 Bp Deletion in Patients with Hepatocellular Carcinoma. Balkan J. Med. Genet..

[146] Zhang Y., Ma Y., Bu D., Liu H., Xia C., Zhang Y., Zhu S., Pan H., Pei P., Zheng X. (2015). Deletion of a 4977-bp Fragment in the Mitochondrial Genome Is Associated with Mitochondrial Disease Severity. PLoS ONE.

[147] Lee H.C., Pang C.Y., Hsu H.S., Wei Y.H. (1994). Differential accumulations of 4,977 bp deletion in mitochondrial DNA of various tissues in human ageing. Biochim. Biophys. Acta..

[148] Ambulkar P.S., Chuadhari A.R., Pal A.K. (2016). Association of large scale 4977-bp “common” deletions in sperm mitochondrial DNA with asthenozoospermia and oligoasthenoteratozoospermia. J. Hum. Reprod Sci..

[149] Bahrehmand Namaghi I., Vaziri H. (2017). Sperm mitochondrial DNA deletion in Iranian infertiles with asthenozoospermia. Andrologia.

[150] Ambulkar P.S., Waghmare J.E., Chaudhari A.R., Wankhede V.R., Tarnekar A.M., Shende M.R., Pal A.K. (2016). Large Scale 7436-bp Deletions in Human Sperm Mitochondrial DNA with Spermatozoa Dysfunction and Male Infertility. J. Clin. Diagn. Res..

[151] Cummins J.M., Jequier A.M., Martin R., Mehmet D., Goldblatt J. (1998). Semen levels of mitochondrial DNA deletions in men attending an infertility clinic do not correlate with phenotype. Int. J. Androl..

[152] St John J.C., Jokhi R.P., Barratt C.L. (2001). Men with oligoasthenoteratozoospermia harbour higher numbers of multiple mitochondrial DNA deletions in their spermatozoa, but individual deletions are not indicative of overall aetiology. Mol. Hum. Reprod..

[153] O’Flaherty C. (2019). Orchestrating the antioxidant defenses in the epididymis. Andrology.

[154] Schneider M., Förster H., Boersma A., Seiler A., Wehnes H., Sinowatz F., Neumüller C., Deutsch M.J., Walch A., Hrabé de Angelis M. (2009). Mitochondrial glutathione peroxidase 4 disruption causes male infertility. FASEB J..

[155] Chabory E., Damon C., Lenoir A., Kauselmann G., Kern H., Zevnik B., Garrel C., Saez F., Cadet R., Henry-Berger J. (2009). Epididymis seleno-independent glutathione peroxidase 5 maintains sperm DNA integrity in mice. J. Clin. Invest..

[156] O’Flaherty C., de Souza A.R. (2011). Hydrogen peroxide modifies human sperm peroxiredoxins in a dose-dependent manner. Biol. Reprod..

[157] Dubuisson M., Vander Stricht D., Clippe A., Etienne F., Nauser T., Kissner R., Koppenol W.H., Rees J.F., Knoops B. (2004). Human peroxiredoxin 5 is a peroxynitrite reductase. FEBS Lett..

[158] Liu Y., O’Flaherty C. (2017). In vivo oxidative stress alters thiol redox status of peroxiredoxin 1 and 6 and impairs rat sperm quality. Asian. J. Androl..

[159] Fisher A.B. (2017). Peroxiredoxin 6 in the repair of peroxidized cell membranes and cell signaling. Arch. Biochem. Biophys..

[160] Fernandez M.C., O’Flaherty C. (2018). Peroxiredoxin 6 is the primary antioxidant enzyme for the maintenance of viability and DNA integrity in human spermatozoa. Hum. Reprod..

[161] Bansal A.K., Bilaspuri G.S. (2010). Impacts of oxidative stress and antioxidants on semen functions. Vet. Med. Int..

[162] Gong S., San Gabriel M.C., Zini A., Chan P., O’Flaherty C. (2012). Low amounts and high thiol oxidation of peroxiredoxins in spermatozoa from infertile men. J. Androl..

[163] Wichmann L., Vaalasti A., Vaalasti T., Tuohimaa P. (1989). Localization of lactoferrin in the male reproductive tract. Int. J. Androl..

[164] Pearl C.A., Roser J.F. (2008). Expression of lactoferrin in the boar epididymis: Effects of reduced estrogen. Domest. Anim. Endocrinol..

[165] Wang P., Liu B., Wang Z., Niu X., Su S., Zhang W., Wang X. (2011). Characterization of lactoferrin receptor on human spermatozoa. Reprod. Biomed. Online.

[166] Hamada A., Sharma R., du Plessis S.S., Willard B., Yadav S.P., Sabanegh E., Agarwal A. (2013). Two-dimensional differential in-gel electrophoresis-based proteomics of male gametes in relation to oxidative stress. Fertil. Steril..

[167] Montecinos V., Guzmán P., Barra V., Villagrán M., Muñoz-Montesino C., Sotomayor K., Escobar E., Godoy A., Mardones L., Sotomayor P. (2007). Vitamin C is an essential antioxidant that enhances survival of oxidatively stressed human vascular endothelial cells in the presence of a vast molar excess of glutathione. J. Biol. Chem..

[168] Nouri M., Ghasemzadeh A., Farzadi L., Shahnazi V., Ghaffari-Novin M. (2008). Vitamins C, E and lipid peroxidation levels in sperm and seminal plasma of asthenoteratozoospermic and normozoospermic men. Iran. J. Reprod. Med..

[169] Micheli L., Cerretani D., Collodel G., Menchiari A., Moltoni L., Fiaschi A.I., Moretti E. (2016). Evaluation of enzymatic and non-enzymatic antioxidants in seminal plasma of men with genitourinary infections, varicocele and idiopathic infertility. Andrology.

[170] Huang C., Cao X., Pang D., Li C., Luo Q., Zou Y., Feng B., Li L., Cheng A., Chen Z. (2018). Is male infertility associated with increased oxidative stress in seminal plasma? A-meta analysis. Oncotarget.

[171] O’Flaherty C., Matsushita-Fournier D. (2017). Reactive oxygen species and protein modifications in spermatozoa. Biol. Reprod..

[172] Piomboni P., Gambera L., Serafini F., Campanella G., Morgante G., De Leo V. (2008). Sperm quality improvement after natural anti-oxidant treatment of asthenoteratospermic men with leukocytospermia. Asian J. Androl..

[173] Akmal M., Qadri J.Q., Al-Waili N.S., Thangal S., Haq A., Saloom K.Y. (2006). Improvement in human semen quality after oral supplementation of vitamin C. J. Med. Food..

[174] Omu A.E., Al-Azemi M.K., Kehinde E.O., Anim J.T., Oriowo M.A., Mathew T.C. (2008). Indications of the mechanisms involved in improved sperm parameters by zinc therapy. Med. Princ. Pract..

[175] Garolla A., Maiorino M., Roverato A., Roveri A., Ursini F., Foresta C. (2005). Oral carnitine supplementation increases sperm motility in asthenozoospermic men with normal sperm phospholipid hydroperoxide glutathione peroxidase levels. Fertil. Steril..

[176] Sigman M., Glass S., Campagnone J., Pryor J.L. (2006). Carnitine for the treatment of idiopathic asthenospermia: A randomized, double-blind, placebo-controlled trial. Fertil. Steril..

